# Immunological Comparison of Native and Recombinant Hen’s Egg Yolk Allergen, Chicken Serum Albumin (Gal d 5), Produced in *Kluveromyces lactis*

**DOI:** 10.3390/nu10060757

**Published:** 2018-06-12

**Authors:** Chamika De Silva, Pathum Dhanapala, Samuel King, Timothy Doran, Mimi Tang, Cenk Suphioglu

**Affiliations:** 1 NeuroAllergy Research Laboratory (NARL), School of Life and Environmental Sciences, Faculty of Science, Engineering and Built Environment, Deakin University, 75 Pigdons Road, Geelong, Victoria 3216, Australia; desilva.chamika@gmail.com (C.D.S.); pathum.dhanapala@gmail.com (P.D.); samuelk@deakin.edu.au (S.K.); 2 Poultry Cooperative Research Centre, P.O. Box U242, University of New England, Armidale, NSW 2351, Australia; Timothy.Doran@csiro.au; 3 Department of Biological Science, Faculty of Applied Sciences, Rajarata University of Sri Lanka, Mihintale 50300, Sri Lanka; 4 Australian Animal Health Laboratory (AAHL), Biosecurity Flagship, Commonwealth Scientific and Industrial Research Organisation (CSIRO), 5 Portarlington Road, Newcomb, VIC 3219, Australia; 5 Department of Allergy and Immunology, Royal Children’s Hospital, 50 Flemington Road, Parkville, VIC 3052, Australia; Mimi.Tang@rch.org.au; 6 Allergy and Immune Disorders, Murdoch Children’s Research Institute, Parkville, Melbourne, Victoria 3052, Australia; 7 Department of Paediatrics, The University of Melbourne, Parkville, Melbourne, Victoria 3052, Australia

**Keywords:** allergy, egg yolk allergens, recombinant allergens, chicken serum albumin

## Abstract

Chicken serum albumin (CSA) is a hen’s egg yolk allergen causing IgE-mediated allergy. The objective of this study was to produce a recombinant version of CSA and compare its IgE reactivity to natural CSA (nCSA). CSA was cloned and expressed as a soluble fraction in the yeast *Kluyveromyces lactis* (*K. lactis*) protein expression system. The gene encoding CSA was amplified with a *C*-terminal hemagglutinin epitope tag by polymerase chain reaction (PCR) and cloned into the pKLAC2 expression vector prior to transforming into *K. lactis*. Recombinant CSA (rCSA) was purified by immunoprecipitation. Twenty-one patients allergic to hen’s egg white were examined for sensitisation against nCSA. 38% of patients were found to be sensitised to CSA based on Western immunoassay. Immunoglobulin E (IgE) binding capacity of rCSA and nCSA was analysed by ELISA using sera from patients sensitised to CSA. Levels of IgE-binding were similar for both the recombinant and the natural CSA, indicating the existence of similar epitopes. rCSA produced in this study is a potential candidate to be used in component-resolved diagnosis (CRD) of egg yolk allergy. The usefulness of rCSA in CRD of egg yolk allergy warrants further characterisation using sera from patients with allergy to hen’s egg yolk in future studies.

## 1. Introduction

Hen’s (*Gallus domesticus*) egg is a popular protein source for humans throughout the world. Egg white in relation to food allergy has been well investigated, unlike the egg yolk. In Australia, the egg white is reported to cause immunoglobulin E (IgE)-mediated hypersensitivity in 9% of infants [1]. The study by Okada et al. estimated that 9.1% of children with hen’s egg allergy were positive for egg yolk slightly contaminated with egg white, based on oral food challenge (OFC) [2]. Two allergens, known as chicken serum albumin (Gal d 5) and yolk glycoprotein 42 (YGP42) (Gal d 6), have been identified from the hen’s egg yolk [3,4]. *In vitro* sensitisation to those egg yolk allergens in children diagnosed with egg allergy have been clearly demonstrated in recent studies [5,6]. However, understanding of allergy to hen’s egg yolk in the clinical setting is very limited and there is a lack of well-defined reagents for diagnosis of egg yolk allergy. Therefore, people with allergy to hen’s egg yolk may not be detected and left with symptoms that are not recognized, thus emphasising the importance of this study. Furthermore, it has been shown that classifying infants with hen’s egg allergy into egg yolk tolerant and egg yolk reactive with slight egg white contamination is useful for predicting the natural course of egg allergy in early childhood [2]. Therefore, accurate diagnosis of egg white and egg yolk allergy is crucial in better management of egg allergy and avoiding unnecessary dietary restrictions.

Skin prick testing (SPT) and food-specific immunoglobulin E (sIgE) levels are excellent tools for diagnosis of food allergies. However, the use of crude allergen extracts reduces the reliability of those tests since they often contain a mixture of allergenic and nonallergenic components. The difficulties associated with crude allergen extracts can be easily overcome by the use of component-resolved diagnosis (CRD) [7]. In CRD, specific allergens are used for measurement of sIgE [8]. The performance of CRD is even more enhanced with the use of well-characterized, recombinant-based allergens, instead of purified natural allergens. Recombinant allergens can be produced to mimic the properties of their natural counterparts and are already used in CRD of allergies [9].

To the best of our knowledge, there are no reports of recombinant production of hen’s egg yolk allergen chicken serum albumin (CSA). In this study, for the first time, we present the recombinant production of hen’s egg yolk allergen CSA. CSA, also known as α-livetin, is a partially heat-labile allergen implicated in bird-egg syndrome [4]. The importance of CSA as a food allergen is demonstrated by several studies. A cross-sectional survey on IgE reactivity to various allergens reported that 0.14% of subjects had IgE against CSA. Another study reported the percentage of IgE sensitisation to CSA to be as high as 20% [10,11].

The main objective of this study was to produce a recombinant version of CSA and characterize its IgE-binding capacity in comparison to natural CSA (nCSA). Although CSA is an egg yolk allergen, the IgE-binding assays were performed using sera from children with medically diagnosed egg white allergy due to the unavailability of sera from children with egg yolk allergy. Furthermore, the cohort of patients used in the study have not been tested for sensitization or clinical reactivity against hen’s egg yolk. As a result, these patients may or may not have sensitization to egg yolk allergens. In this study, we report successful cloning and expression of recombinant CSA (rCSA) as a soluble fraction in *K. lactis* yeast strain. Immunological comparison of rCSA and nCSA was carried out qualitatively and quantitatively, using sera from children diagnosed with allergy to hen’s egg white. The results of immunoassays confirmed that the rCSA produced in this study is similar to nCSA in terms of IgE-binding reactivity, indicating the preservation of relevant allergenic epitopes.

## 2. Materials and Methods

### 2.1. Human Patients’ Sera

Sera from 21 patients sensitised to hen’s egg white were obtained from the Royal Children’s Hospital (Melbourne, VIC, Australia). Serum levels of egg white sIgE are shown in Table 1. This study was conducted in compliance with the National Statement on Ethical Conduct in Human Research (2007) with approval from Deakin University Faculty of Science, Engineering and Built Environment Human Ethics Advisory Group (HEAG), with project approval number of STEC-34-2013-DHANAPALA.

### 2.2. Natural Allergen Extract

Lyophilised nCSA powder was purchased from Equitech-Bio, Inc. (Kerrville, TX, USA) and prepared at a concentration of 10 mg/mL in 1× phosphate buffered saline (PBS). nCSA was purchased at the highest available purification level (≥96% purity).

### 2.3. Strains, Vectors, and Growth Conditions

*Escherichia coli* (*E. coli*) strain NEB^®^ 5-alpha F’ *I*^q^ (New England Biolabs Inc., Ipswich, MA, USA) was used as the cloning host and was grown in Luria-Bertani (LB) medium supplemented with 50 mg/mL ampicillin at 37 °C. *K. lactis* strain GG799 (New England Biolabs Inc., Ipswich, MA, USA) was used as the host strain for the secretion of rCSA. *K. lactis* were grown in either YPGal medium (1% yeast extract, 2% peptone, 2% galactose) or YPGlu medium (1% yeast extract, 2% peptone, 2% glucose) at 30 °C. The *K. lactis* integrative expression vector pKLAC2 (New England Biolabs Inc., Ipswich, MA, USA) contains the *Aspergillus nidulans* acetamidase gene (*amdS*), which allows growth on nitrogen-free minimal medium containing acetamide. Selection of *K. lactis* cells transformed with pKLAC2 vectors was performed by growth on yeast carbon base (YCB) agar medium with 5 mM acetamide at 30 °C.

### 2.4. Synthesis of the CSA Gene and the Construction of the pKLAC2-CSA Expression Vector

The CSA gene was amplified through polymerase chain reaction (PCR) using the QIAGEN Fast Cycling PCR kit (Qiagen, Hilden, Germany). pTrcHis A-CSA expression vector was used as the template DNA (previously constructed in our laboratory). Primers used for amplification of the CSA gene were based on the sequence published on the National Centre for Biotechnology Information (NCBI) web site (NCBI accession number: NM_205261.1). The sequence coding for the mature CSA protein was amplified with the forward primer 5′-CGCCTCGAG*AAAAGA*TTTGCTCGTGATGCAGAGCACAAGAG-3′ and reverse primer 5′-CGCGCGGCCGCTTA**TGCATAATCTGGAACATCATATGGATA**AGCACCAATTCCTAATGTGGCTCTGC-3′. XhoI and NotI restriction enzyme sites were incorporated into 5′ ends of forward and reverse primers, respectively (restriction sites underlined in the primer sequences). Forward primer contains a Kex protease cleavage site immediately downstream of the XhoI restriction site (nucleotides in italics). The reverse primer contains sequence for *C*-terminal hemagglutinin (HA) epitope (nucleotides in bold) and a TTA stop codon immediately downstream of the NotI restriction site. Both primers contain CGC extensions at the 5′ ends. Thermal cycler conditions were: 5 min at 95 °C for activation of HotStart DNA polymerase, 35 cycles of 5 s denaturation at 96 °C, 5 s annealing at 60 °C, 5.34 min of extension at 68 °C, and a final extension for 1 min at 72 °C. The PCR product was analysed on E-Gel^®^CloneWell^™^ agarose gels with 0.8% SYBR^®^ Safe DNA gel stain (ThermoFisher Scientific, Waltham, MA, USA). The PCR-amplified mature CSA gene was cloned into the XhoI and NotI sites of the pKLAC2 vector in frame with the α-mating factor (α-MF) secretion leader sequence to yield pKLAC2-CSA construct. The pKLAC2-CSA was then chemically transformed into competent NEB^®^ 5-alpha F’ *I*^q^ *E. coli* cells. Transformed cells were grown overnight on LB agar with 50 mg/mL ampicillin at 37 °C. Plasmids from positive *E. coli* transformants were isolated by QIAprep^®^ Spin Miniprep kit (Qiagen, Hilden, Germany) and sequenced (Micromon, Monash University, Clayton, Australia) to confirm successful insertion of the CSA sequence into pKLAC2.

### 2.5. Transformation of K. lactis

pKLAC2 containing the CSA gene was digested with *SacII* restriction enzyme in order to produce the linear expression cassette. Digested DNA was then desalted using QIAquick^®^ PCR Purification kit (Qiagen, Hilden, Germany). A total of 1 µg of linearised DNA in a volume less than 15 µL was used to transform chemically competent *K. lactis* cells according to manufacturer’s instructions. *K. lactis* cells harboring successfully integrated pKLAC2 DNA containing CSA were obtained by growth on YCB agar medium supplemented with 5 mM acetamide at 30 °C for 3 to 4 days.

### 2.6. Identification of Multicopy Integrants

*K. lactis* transformants with multicopy integrants were identified through whole cell PCR. Patch plates were made on YCB agar supplemented with 5 mM acetamide using transformed *K. lactis* cells. *K. lactis* cells from an area approximately 1 mm^2^ were harvested by scraping with a pipette tip and resuspended in 25 µL of 1 M sorbitol containing 10 mg/mL lyticase (Sigma-Aldrich, St. Louis, MO, USA). The cells were mixed by vortexing and incubated at 30 °C for 1 h. The lyticase treated cells were lysed in a thermocycler by incubating at 98 °C for 10 min. Then, lyticase treated cells were used as the template in a PCR reaction with integration primer 2 (5′-ATCATCCTTGTCAGCGAAAGC-3′) and integration primer 3 (5′-ACCTGAAGATAGAGCTTCTAA-3′) in a 100 µL total reaction volume. The thermal cycler conditions were: 30 cycles with 30 s at 94 °C, 30 s at 50 °C, and 3 min at 72 °C and a final incubation at 72 °C for 10 min. The whole cell PCR products were analysed on E-Gel^®^CloneWell^™^ agarose gels with 0.8% SYBR^®^ Safe DNA gel stain.

### 2.7. Time Course of Expression and Detection of Secreted Proteins

Transformed *K. lactis* cells containing multicopy integrants were screened for their ability to secrete rCSA in YPGal medium. For expression, a *K. lactis* clone bearing multicopy integrants was grown in 5 mL of YPGal medium at 30 °C with shaking at 250 rpm for 7 days. Culture supernatants were harvested by centrifugation at 4700× *g* for 3 min every 24 h to analyse secretion of rCSA. The saved culture supernatants were examined on a 4–20% Novex^®^ Tris-Glycine gel by sodium dodecyl sulphate gel electrophoresis (SDS-PAGE) under reducing and denaturing conditions. The secretion of rCSA protein fused to HA-epitope was confirmed by Western immunoassay using the monoclonal anti-HA antibody produced in mouse (monoclonal HA-7, ascites fluid) (Sigma-Aldrich, St. Louis, MO, USA). Furthermore, nCSA was analysed on SDS-PAGE under reducing and denaturing conditions.

### 2.8. Purification of rCSA

Purification of rCSA from 6-day spent culture media was performed using Pierce^TM^ Magnetic HA-Tag IP/Co-IP kit (ThermoFisher Scientific, Waltham, MA, USA). 25 µL of Pierce^TM^ Anti-HA Magnetic beads were first washed with Pierce^TM^ IP Lysis/Wash Buffer (pH 7.4, 0.025 M Tris, 0.15 M NaCl, 0.0001 M EDTA, 1% NP40, 5% glycerol) and incubated with 300 µL of rCSA containing culture supernatant for 30 min with mixing at room temperature. Beads were collected using a magnetic stand and the unbound sample was saved for analysis. The collected beads were washed with IP Lysis/Wash Buffer and ultrapure water. 100 µL of Elution Buffer (pH 2.0) was added to the beads and incubated for 10 min with mixing at room temperature. Beads were separated using a magnetic stand and the supernatant containing rCSA was saved. To neutralise the low pH, 15 µL of Neutralization Buffer (pH 8.5) was added. The purified fractions were analysed by SDS-PAGE and Western immunoblotting under reducing and denaturing conditions. The concentration of purified rCSA was measured using Pierce^TM^ BCA Protein Assay-Test Tube protocol (ThermoFisher Scientific, Waltham, MA, USA).

### 2.9. Western Dot-Blot Assay of nCSA

3 µg of nCSA in a total volume of 100 µL of 1× PBS was spotted onto a nitrocellulose membrane using the Bio-Dot Microfiltration apparatus (Bio-Rad, Hercules, CA, USA). Following blocking, nCSA spots were incubated with sera from 21 patients (diluted 1:10) sensitised to hen’s egg white and sera from 2 healthy (control) patients (diluted 1:10) overnight with gentle agitation at room temperature. After washing, all serum pre-incubated spots were incubated with monoclonal antihuman IgE antibody produced in goat and labelled with alkaline phosphatase (diluted 1:2000) (Sigma-Aldrich, St. Louis, MO, USA). The signal was developed by incubating the nitrocellulose membrane with 5-bromo-4-chloro-3′ indolyphosphate p-toluidine/nitro-blue tetrazolium chloride (BCIP/NBT) chromogenic substrate (ThermoFisher Scientific, Waltham, MA, USA).

### 2.10. Dot-Blot Analysis of rCSA

The dot-blot analysis of rCSA was conducted using the Bio-Dot Microfiltration apparatus according to the protocol described in Section 2.9. One rCSA spot on the membrane was probed with pooled serum (diluted 1:5 in 1% nonfat dry milk in 1× PBS) from 8 patients sensitised to nCSA, as identified in the previous dot-blot assay. As a negative control, two rCSA spots on the membrane were probed with sera from 2 healthy individuals. After washing, all serum pre-incubated spots were incubated with monoclonal antihuman IgE antibody produced in goat and labelled with alkaline phosphatase (diluted 1:2000). The signal was developed by incubating the nitrocellulose membrane with BCIP/NBT chromogenic substrate.

### 2.11. Indirect ELISA for Quantification of sIgE against rCSA and nCSA

1 µg of nCSA and rCSA in 100 µL of coating buffer (100 mM bicarbonate/carbonate coating buffer, pH 9.6) were allowed to coat wells in an ELISA microtiter plate overnight at 4 °C. After triple washing with 0.05% phosphate buffered saline with Tween 20 (PBST), all protein-coated wells were blocked with 1% nonfat dry milk in 1× PBS for 2 h at room temperature. The plates were triple washed with 0.05% PBST. nCSA- and rCSA-coated wells were then incubated with sera (diluted 1:10 in 1% nonfat dry milk in 1× PBS) from patients 4, 5, 6, 8, 10, 11, 14, 18, and one healthy control (diluted 1:10 in 1% nonfat dry milk in 1× PBS) overnight at 4 °C. Samples were conducted in triplicates. After triple washing the wells with 0.05% PBST, all wells were incubated with monoclonal antihuman IgE antibody produced in goat and labelled with alkaline phosphatase (diluted 1:1000 in 1% nonfat dry milk in 1× PBS) for 2 h at room temperature. Following triple washing with 0.05% PBST, 200 µL of alkaline phosphatase Yellow (*p*-nitrophenylphosphate; pNPP) liquid substrate (Sigma-Aldrich, St. Louis, MO, USA) was added to the wells and incubated at 37 °C until colour development was observed. The colour development was terminated by adding 3 M NaOH (50 µL per well) and the plate was read at 405 nm using a plate reader.

## 3. Results

### 3.1. Synthesis of pKLAC2-CSA Construct and Expression of rCSA by K. lactis

The PCR-amplified CSA gene was cloned into the pKLAC2 expression vector and transformed into *E. coli*. Positive recombinants were identified by sequencing plasmid DNA of transformed *E. coli* grown under ampicillin selection. DNA sequencing results confirmed the presence of the mature CSA gene and the in-frame cloning into the pKLAC2 vector. The pKLAC2-CSA recombinant vectors were then transformed chemically into competent *K. lactis*.

**Figure 1:**
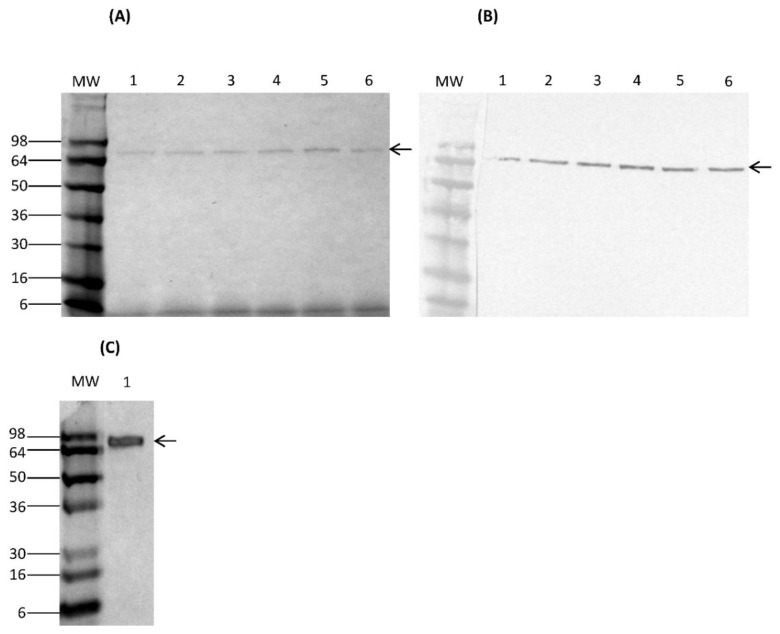
Time course of expression of *K. lactis* culture supernatant fractions containing secreted rCSA by (**A**) SDS-PAGE and (**B**) Western immunoblot analysis. Lane 1–6: galactose-induced culture supernatants from day 2–7. (**C**) SDS-PAGE analysis of purified nCSA. The arrows indicate the positions of both rCSA and nCSA at 69 kDa. MW: molecular weight marker SeeBlue^®^ Pre-Stained Protein Standard. MW: molecular weight marker; rCSA: recombinant chicken serum albumin; SDS-PAGE: sodium dodecyl sulphate gel electrophoresis; nCSA: natural chicken serum albumin.

*K. lactis* transformants were selected by growth on YCB agar containing 5 mM acetamide. Strains containing tandem copies of pKLAC2 expression cassette were confirmed by whole cell PCR. Analysis of PCR products on agarose gels revealed an amplification product of approximately 2.3 kb (results not shown). Transformants were further screened for their ability to secrete rCSA in YPGal medium. SDS-PAGE and Western immunoblot analysis of culture supernatants showed a predominant band at approximately 69 kDa, corresponding to the size of nCSA (results not shown). The results for time course of expression and SDS-PAGE analysis of rCSA are shown in Figure 1. The time course of expression analysis of transformed *K. lactis* cells exhibited an optimum expression of rCSA after 6 days (Figure 1A, B). The SDS-PAGE of nCSA also showed a single predominant band at approximately 69 kDa (Figure 1C).

**Figure 2:**
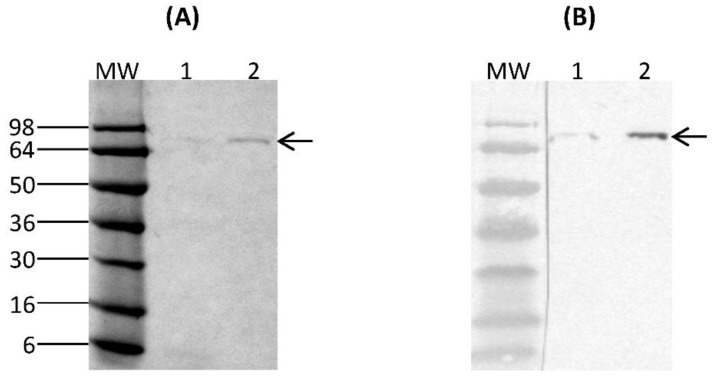
Immunoprecipitation analysis of purified rCSA fractions by (**A**) SDS-PAGE and (**B**) Western immunoblotting. Lane 1: unbound sample, Lane 2: elution containing rCSA. Arrows indicate the rCSA. MW: molecular weight marker SeeBlue^®^ Pre-Stained Protein Standard.

Expression of a recombinant protein fused to a short polypeptide tag such as HA epitope provides the means to purify the fusion protein using immunoprecipitation. The SDS-PAGE analysis of immunoprecipitation-purified rCSA fraction showed a predominant band, approximately at 69 kDa with no visible contaminant *K. lactis* proteins (Figure 2A). The authenticity of purified rCSA fusion protein was confirmed by probing with monoclonal anti-HA antibody produced in mouse, which showed a predominant band at approximately 69 kDa (Figure 2B). Furthermore, in all SDS-PAGE and Western immunoblot analysis of culture supernatants, no proteolytic degradation of rCSA was observed. The rCSA concentration of 6-day spent culture media was 54 μg/mL.

### 3.2. IgE-Binding Capacity of nCSA and rCSA

**Figure 3:**
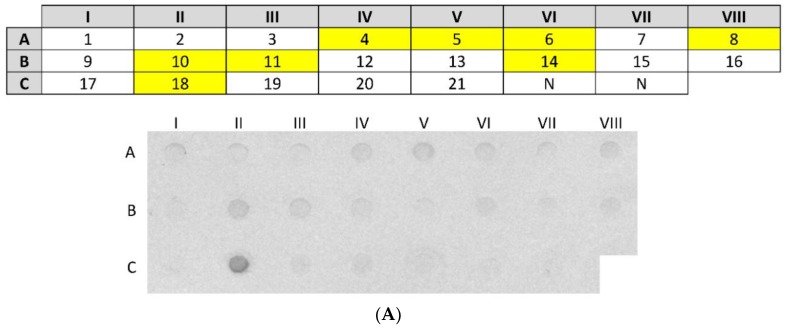

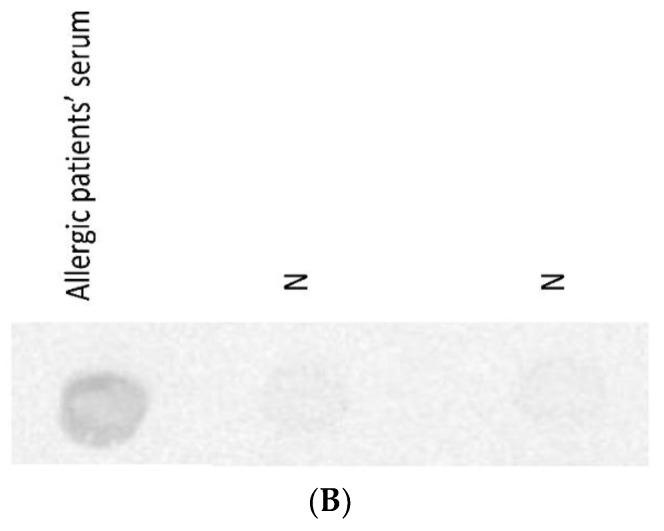
(**A**) IgE reactivity to nCSA in patients sensitised to hen’s egg white determined by dot blot. The grid above shows the patient ID described in Table 1 corresponding to each spot. nCSA-sensitised patients are highlighted in the grid. Dots denoted by N are sera from nonallergic (control) subjects. A to C represent row identity and I to VIII represent column identity in the grid and the corresponding Western dot blot; (**B**) IgE reactivity to rCSA of sera from patients sensitised to nCSA described in Figure 3 by Western dot-blot analysis. Dots denoted by N are sera from nonallergic healthy (control) subjects.

Sera from 21 patients sensitised to hen’s egg white were tested with dot-blot analysis to examine the presence and level of IgE binding to nCSA (Figure 3A). Dot-blot analysis showed IgE reactivity to nCSA in 38% (8 out of 21) of egg-white-sensitised patients. None of the healthy controls showed any visible IgE reactivity against nCSA. Patient 18 showed very strong IgE reactivity against nCSA when compared to control and other nCSA-sensitised patients. The remaining nCSA-reactive patients exhibited only low levels of IgE binding to nCSA. Allergen-sIgE reactivity against rCSA was also confirmed by dot-blot analysis of rCSA using pooled serum from nCSA-sensitised patients (Figure 3B).

**Figure 4:**
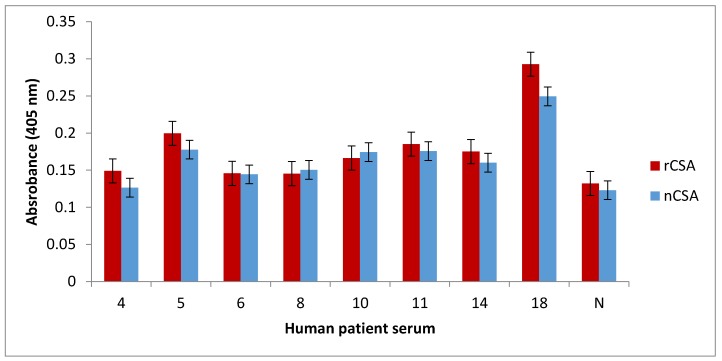
Comparison of the binding activity of human sIgE against recombinant CSA and natural CSA. N denotes serum from healthy subjects and numbers denote different patients’ sera sensitised to nCSA. Data are average of triplicate mean +/− SEM. sIgE: specific immunoglobulin E; SEM: standard error of mean.

An indirect ELISA assay (Figure 4) was performed to comparatively quantify the levels of allergen-sIgE directed against nCSA and rCSA using sera from patients sensitised to nCSA, identified by the previous dot-blot experiment (Figure 3A). Patient 18 had the highest amount of IgE binding against both natural and recombinant CSA, while other sensitised patients showed lower levels of allergen-sIgE binding. Furthermore, IgE-binding activity was very similar for both natural and recombinant CSA in all patients.

## 4. Discussion

The main objective of this study was to produce a recombinant version of hen’s egg yolk allergen CSA and analyse the human IgE-binding capacity in comparison to nCSA using sera from patients sensitised to hen’s egg white. Sera from patients with allergy to hen’s egg yolk would have been ideal for the immunological study since CSA is an egg yolk allergen. Patients with allergy to egg white may or may not develop allergic reactions to egg yolk allergens. Therefore, egg-white-sensitised patients may or may not have sensitisation against egg yolk allergen CSA. Our findings suggest that rCSA produced by *K. lactis*is is IgE-reactive and is very similar to nCSA in terms of IgE-binding capacity. Furthermore, comparative immunoassay results suggest that some patients with allergy to hen’s egg white may have the risk of allergic reactions to hen’s egg yolk as well due to sensitisation against CSA.

*K. lactis* was engineered to uptake multiple tandem copies of the expression fragment in order to increase production of recombinant protein [12,13]. To increase the level of rCSA production, we only screened for positive *K. lactis* transformants containing multicopy integrants of pKLAC2 expression cassette, using whole cell PCR technique. In this strategy, only strains containing multiple copies of pKLAC2 expression cassette will produce an amplicon with a size of 2.3 kb. However, this technique is unable to indicate the number of integrated expression vector fragments in the cells [13].

Analysis of galactose-induced *K. lactis* culture supernatants with SDS-PAGE and Western immunoblot assays confirmed the presence of rCSA at the expected size. These data suggest that the pKLAC2-containing CSA gene was successfully integrated into the *K. lactis* genome at the *LAC*4 locus, which drives protein expression. This result indicates that cloning of the CSA gene into the pKLAC2 expression vector had been done in-frame with the α-MF domain, which directs the secretion of the rCSA. Furthermore, there weren’t any visible *K. lactis-*derived proteins in the culture supernatants. Therefore, lack of detectable amounts of native host proteins served as the first step of purification.

The expression of a fusion protein with an HA-epitope tag provides the means to selectively isolate proteins through immunoprecipitation, in addition to serving as an identification tag. The rCSA was purified to homogeneity by immunoprecipitation using anti-HA magnetic beads. There were no visible *K. lactis*-derived proteins on SDS-PAGE, indicating a high level of purity. The Western immunoblot assay of purified rCSA indicated that most of the anti-HA magnetic beads were able to capture the target rCSA proteins without any cross-reactivity with native *K. lactis* host proteins. A major concern in recombinant protein production is protein degradation due to proteolysis [14]. Our results confirmed that there was no proteolytic degradation of rCSA, which is important for the preparation of relevant and standardized extracts for the accurate diagnosis and treatment of specific allergies.

The dot-blot immunoassay showed that 38% (8 out of 21) of patients had IgE sensitization against nCSA. The main limitation in this study is the use of sera from patients diagnosed with egg white allergy rather than from patients diagnosed with allergy to egg yolk. Therefore, in our study, we are not able to fully characterise the IgE-reactivity pattern of rCSA and its clinical relevance to IgE-mediated egg yolk hypersensitivity. However, patients who had sIgE against nCSA may or may not be clinically reactive towards hen’s CSA, since the presence of allergen sIgE antibodies is not a definitive marker of clinical reactivity. Nevertheless, presence of such antibodies may have predictive value, as the level of sIgE against food allergens has been shown to correlate with increased likelihood of clinical allergy [15].

IgE-binding capacity between recombinant and corresponding natural allergens may be different. Therefore, it is crucial to immunologically analyse the IgE-binding capacity of native and recombinant allergens, prior to conducting further studies [14]. In this study, we comparatively analysed the degree of IgE binding to recombinant and natural CSA. We showed that recombinant and natural CSA have very similar levels of IgE binding in all of the patients tested. Furthermore, the dot-blot analysis of rCSA confirmed that it is IgE-reactive. These results together suggest that rCSA produced by *K. lactis* is recognisable by the human immune system and bears similar epitopes to that of nCSA.

## 5. Conclusions

In conclusion, this study presents the successful production of IgE-reactive rCSA using *K. lactis* yeast strain. We have shown that rCSA has similar IgE reactivity to nCSA using sera from egg-white-sensitised patients. Therefore, rCSA produced in this study can be considered as a potential candidate for the future use in CRD of egg yolk allergy. However, this study provides the necessary platform for future studies aimed at producing and establishing reagents for diagnosis of egg yolk allergy. Future studies would ideally include immunological analysis of the rCSA using serum from patients diagnosed with allergy to hen’s egg yolk. Furthermore, rCSA should be subjected to basophil activation tests and T cell proliferation assays to evaluate its level of immunological reactivity compared to nCSA. Recombinant DNA technology provides a means for development of hypoallergenic derivatives of CSA, if desired, and the availability of well-defined recombinant allergens can immensely benefit diagnosis and therapy in the future. Finally, it is important to note here that it is pivotal to include testing for egg yolk allergens when assessing patients with possible egg allergy, since patients with egg allergy have been shown to produce allergen sIgE against proteins from both the egg white and the egg yolk.

## Figures and Tables

**Table 1:** Serum levels of specific immunoglobulin E (sIgE) against egg white determined by ImmunoCAP (Phadia).

Patient ID	IgE Level (kU/L) against Hen’s Egg White
1	1.25
2	16.5
3	6.89
4	1.78
5	0.47
6	2.21
7	3.62
8	2.08
9	0.32
10	4.13
11	1.72
12	93.5
13	18.5
14	0.18
15	1.21
16	2.58
17	1.05
18	13.6
19	9.26
20	28.8
21	1.32

## Abbreviations

nCSA	Natural chicken serum alumin
rCSA	Recombinant chicken serum albumin
IgE	Immunoglobulin E
sIgE	Specific IgE
ELISA	Enzyme-linked immunosorbent assay
CRD	Component resolved diagnosis
